# Diagnostic accuracy of left atrial function and strain for differentiating between acute and chronic myocardial infarction

**DOI:** 10.1186/s12872-023-03254-3

**Published:** 2023-04-28

**Authors:** Xiaofeng Jiang, Yi Yan, Zhi Yang, Miao Wen, Yitian Long, Bing Fu, Jian Jiang

**Affiliations:** 1 Department of Radiology, The First Affiliated Hospital of Nanchang University, Nanchang, 330000 China; 2 Department of Pain, The First Affiliated Hospital of Nanchang University, Nanchang, China; 3 The Fifth People’s Hospital of Chengdu, Chengdu, China

**Keywords:** Cardiac magnetic resonance (CMR), Tissue tracking, Acute myocardial infarction (AMI), Chronic myocardial infarction (CMI), LVEF, Strain, LGE, Left atrial function, Left atrial strain

## Abstract

**Background:**

The cardiac magnetic resonance tissue tracking (CMR-TT) technique was used to obtain left atrial strain and strain rate in patients with myocardial infarction (MI) and to evaluate the utility of this technique in the quantitative assessment of myocardial infarction for distinguishing acute from chronic myocardial infarction.

**Methods:**

We retrospectively analyzed 36 consecutive patients with acute myocardial infarction (AMI) and 29 patients with chronic myocardial infarction (CMI) who underwent CMR and 30 controls. Left atrial (LA) and ventricular functions were quantified by volumetric, and CMR-TT derived strain analysis from long and short left ventricular view cines. Receiver Operating Characteristics (ROC) analysis was used to determine the diagnostic accuracy of CMR-TT strain parameters for discriminating between acute and chronic myocardial infarction.

**Results:**

AMI and CMI participants had impaired LA reservoir function, conduit function and LA booster pump dysfunction compared to the controls. LA strain was more sensitive than LV global strain for the assessment of the MI stage. Peak late-negative SR yielded the best areas under the ROC curve (AUC) of 0.879, showing differentiation between acute and chronic myocardial infarction of all the LA strain parameters obtained. The highest significant differences between chronic myocardial infarction and normal myocardium were also found in the LV strain (*p* < 0.001) and LA functional parameters (*p* < 0.001), but there was no difference between AMI and normals.

**Conclusions:**

CMR-TT-derived LA strain is a potential and robust tool in demonstrating impaired LA mechanics and quantifying LA dynamics, which have high sensitivity and specificity in the differential diagnosis of acute versus chronic myocardial infarction. Their use is thus worth popularizing in clinical application.

Myocardial infarction (MI) is caused by a substantial decrease or complete cessation of blood flow to a portion of the myocardium, causing the damaged tissue to be replaced with a fibrotic scar produced by fibroblasts and myofibroblasts. Subsequent compensatory fibrosis of the injured myocardium is prone to systolic and diastolic dysfunction [1]. MI is a significant worldwide burden that accounts for the leading causes of death [2]. Differentiation between acute (AMI) and chronic myocardial infarction (CMI) is clinically significant for patient treatment and follow-up in cases of preexisting CMI, and limited possibility of localizing the acute lesion through ECG or coronary angiography [3]. Late gadolinium enhancement(LGE) is the gold standard for measuring the region and size of myocardial infarction [4]. However, its use in discriminating AMI from CMI is limited.

Cardiovascular magnetic resonance tissue tracking (CMR-TT) technology has been widely used in clinical research and practice for a variety of heart diseases in recent years, which is an approach to assess myocardial deformation from steady-state free precession (SSFP) cine CMR by the tracking of tissue voxel motion [5]. CMR-TT can quantify the early deformation of the left atrium (LA) and ventricle without using contrast agents [6]. It provides a higher spatial resolution and a more extensive field of view, which can reflect the functional characteristics of myocardial tissue more sensitively [7, 8]. Speckle tracking echocardiography (STE) was the first modality to assess atrial strain [9], but it has several limitations, including a suboptimal field of view in the setting of poor acoustic windows and high interobserver variability [10]. Conversely, CMR is a mainstay in the non-invasive assessment of LA volume and function. Several studies have demonstrated that LA deformation detected by CMR-FT can allow for an accurate and reproducible analysis of the LA function [11]. Recently, the importance of LA function and structure has been increasingly acknowledged and led to the introduction of atrial cardiomyopathy as an independent entity [12]. LA modulates LV diastolic filling and cardiac performance during hemodynamic stress or exertion by reservoir, conduit, and booster pump functions [13, 14]. Greater atrial volume is commonly associated with cardiovascular diseases, such as ischemic heart disease, valvular heart disease, and heart failure (e.g., dilated cardiomyopathy) [15]. However, beyond LA volume following AMI, strain and strain rate may be sensitive indicators of left atrial function that have been used to predict cardiovascular mortality [16, 17].

In this study, we aim to compare the impact on LA function between patients with AMI and CMI, as assessed through simultaneous LA and LV structural and functional analyses using CMR-TT, and explore the capability of LA function and strain for distinguishing AMI from CMI.

## Materials and methods

### Study participants

Between January 2016 and December 2021, 36 consecutive patients with AMI and 29 patients with CMI who received CMR examination at our hospital were retrospectively recruited in this study, with an equal gender and age distribution. Inclusion criteria for patients with AMI were as follows: (1) the patients had to have a first-time AMI with an identified culprit coronary vessel;(2) All patients received successful reperfusion therapy by percutaneous coronary intervention. AMI was diagnosed by history, electrocardiographic changes, cardiac biomarker abnormalities, and coronary angiography following the consensus of the American College of Cardiology and the European Society of Cardiology [18]. The inclusion criteria for CMI: (1) severe chest pain with a duration longer than 30 min; (2) definite MI history and the patients were treated with reperfusion therapy utilizing primary percutaneous coronary intervention longer or equal to 6 months; (3) confirmed of coronary artery stenosis by digital subtraction angiography or computed tomography angiography examination. Exclusion criteria were (1) severe chronic kidney disease; (2) known cardiomyopathy; (3) prior cardiac surgery, severe claustrophobia; (4) gadolinium allergy, and (5) ferrous metallic implants. Twenty-nine healthy subjects without any cardiovascular disease symptoms and with normal electrocardiogram (ECG) results were included. This study was approved by our hospital’s committee, and written informed consent was waived because it is a retrospective study.

### Cardiac MRI protocol

Study participants underwent cardiac MRI with clinical 1.5T scanners. We obtained tissue characterization and function in two- and four-chamber long-axis views and short-axis images of the left ventricle with cardiac vector ECG and respiratory gating for scanning. The scan parameters of cine images of Philips Achieva were: TR/TE = 3.38/1.69 ms, FOV: 69.8 cm×32 cm; layer thickness 8 mm, layer interval 2 mm, matrix 192 × 180, and flip angle 60°. Gadopentetate glucosamine (Gd-DTPA) was used as the contrast agent at a dose of 0.2 mmol/kg and an infusion rate of 2.0 ml/s. After the contrast agent was injected, 20 ml of saline was injected at the same rate, and delayed enhancement scans of the heart were done 15 min after the contrast agent was injected intravenously. The delayed enhancement scan parameters were TR/TE = 6.12/3.00 ms, layer thickness 8 mm, layer spacing 2 mm, matrix 152 × 200.

### CMR analysis

All CMR analysis was performed offline using commercially available software (CVI42 version 5.12.4, Circle Cardiovascular Imaging, Calgary, Canada). The left ventricular function parameters were performed by drawing out the endo- and epicardial border on LV short-axis cine images (papillary muscles were excluded). The software automatically tracked the displacement of the endocardium and epicardium to quantify the left ventricular myocardium movement. And then, the LV end-diastolic volume (LVEDV), LV end-systolic volume (LVESV), LV ejection fraction (LVEF), LV cardiac output (CO), LV cardiac index (CI), and LV mass (LVM) were well obtained. LV strain values were acquired by autonomously tracking the deformation of myocardial motion during the cardiac cycle, including left ventricle global peak strain radial (LV-GPSr), left ventricle global peak strain circumferential (LV-GPSc) and left ventricle global peak strain longitudinal (LV-GPSl). LGE was taken as the golden standard to identify infarcted segments. Infarct size was defined as the hyper-enhanced area with a signal intensity threshold ≥ 5 standard deviations (SD) above the remote myocardium’s mean signal intensity.

**Fig. 1:**
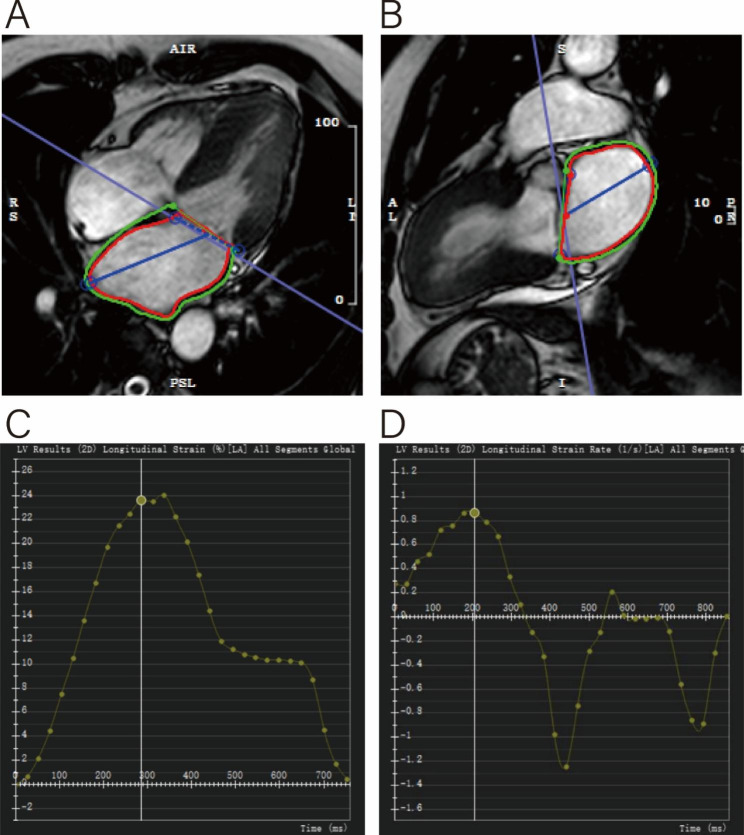
Male, 56 year, LA measurements by CMR feature tracking. (A and B) LA longitudinal strain in the four- and two-chamber views at end-diastole. (C and D) The LA strain and strain rate curve. Global endocardial LA strain and strain rate values were recorded. εs, reservoir strain; εa, booster strain; εe, conduit strain; εe = εs-εa. SRs, peak positive strain rate;SRe, peak early-negative SR; SRa, peak late-negative SR.

LA volumetric indices were measured by semi-automated tracing of the LA endocardial and epicardial border in end-systole and end-diastole in long-axis two- and four-chamber views excluding pulmonary veins and the LA appendage. Maximum LA volumes were assessed in ventricular end-systole (LAVmax), at ventricular diastole before atrial contraction (LAVpac), and at late ventricular diastole after atrial contraction (LAVmin). Left atrial fractions were defined as fractional volume changes according to the following equations: LA total EF = (LAVmax − LAVmin) × 100%/LAVmax, LA passive EF = (LAVmax − LAVpre-a) × 100%/LAVmax, LA active EF = (LAVpre-a − LAVmin) × 100%/LAVpre-a. The software automatically derived the strain and strain rate values for each tissue point and was represented as a strain curve from which LA strain and strain rate for each period were recorded (Fig. 1). The following LA global functional parameters were quantitatively analyzed: reservoir function (total ejection fraction [LA total EF], total strain [εs], peak positive strain rate [SRs]), conduit function (passive ejection fraction [LA passive EF], passive strain [εe], peak early-negative SR [SRe]), and booster pump function (active ejection fraction [LA active EF], active strain [εa], late peak negative SR [SRa]). LGE was taken as the golden standard to identify infarcted segments.

Reproducibility.

The intra- and inter-observer variability for the LA volume, strain, and SR measurements were assessed by the intraclass correlation coefficient (ICC) in 20 randomly selected subjects (10 healthy subjects and 10 MI patients). Intra-observer reproducibility was established by the same observer who re-analyzed the same 20 subjects after 1 Month. Inter-observer reproducibility was assessed by two investigators blinded to each other’s results.

### Statistical analysis

SPSS version 26.0 (IBM, Armonk, NY, USA) was used for statistical analyses. Normally distributed continuous variables were verified using Kolmogorov–Smirnov test. Measurement data were expressed as mean ± SD. Categorical variables were compared using Fisher’s exact test or χ2 tests. Comparisons of continuous variables among three groups were performed using one-way analysis of variance (ANOVA), followed by the Tukey or Games-Howell post hoc pairwise comparison test, respectively. Categorical variables were expressed as numbers and percentages. Independent samples t-test was used to compare the global left ventricular strain and Infarct volume in patients with AMI and CMI. The Receiver Operating characteristic Curve (ROC) was used to analyze the value of LA strain and strain rate parameters in identifying AMI and CMI. *P* value < 0.05 indicated a statistically significant difference.

## Results

### Patient characteristics

Initially, 81 patients were included; however, 11 patients were excluded due to poor image quality, and five patients were dropped because no LGE images were available. In the final study cohort, 36 patients with AMI and 29 patients with CMI were included for analysis in this study with sample size- and sex-matched controls. There were no significant differences in gender or body Mass Index among the three groups. The complete baseline characteristics were shown in Table 1.

**Table 1:** Baseline clinical characteristics

	AMI(n = 36)	CMI(n = 29)	Control(n = 30)	*P* value
Clinical baseline				
Age, y	51.00 ± 2.70	61.78 ± 2.19	55.60 ± 2.78	0.087
Male, n(%)	30(83)	23(79)	23(77)	0.792
Height, cm	166.16 ± 1.89	165.06 ± 1.50	162.47 ± 2.54	0.317
Weight, kg	65.85 ± 2.49	65.64 ± 2.36	68.57 ± 4.06	0.880
BMI, (kg/m^2^)	23.90 ± 0.89	24.00 ± 0.71	25.80 ± 1.165	0.405
Diabetes, n(%)	13(36)	12(41)	-	0.664
Hypertension, n(%)	18(50)	14(48)	-	0.890
Hyperlipidemia, n(%)	5(14)	2(6.9)	-	0.447
Smoker, n(%)	30(83)	19(66)	-	0.097

Data are expressed as mean ± standard deviations or percentages in parentheses. There was no significant difference between MI and controls

### Left ventricular structural and functional abnormality

**Fig. 2:**
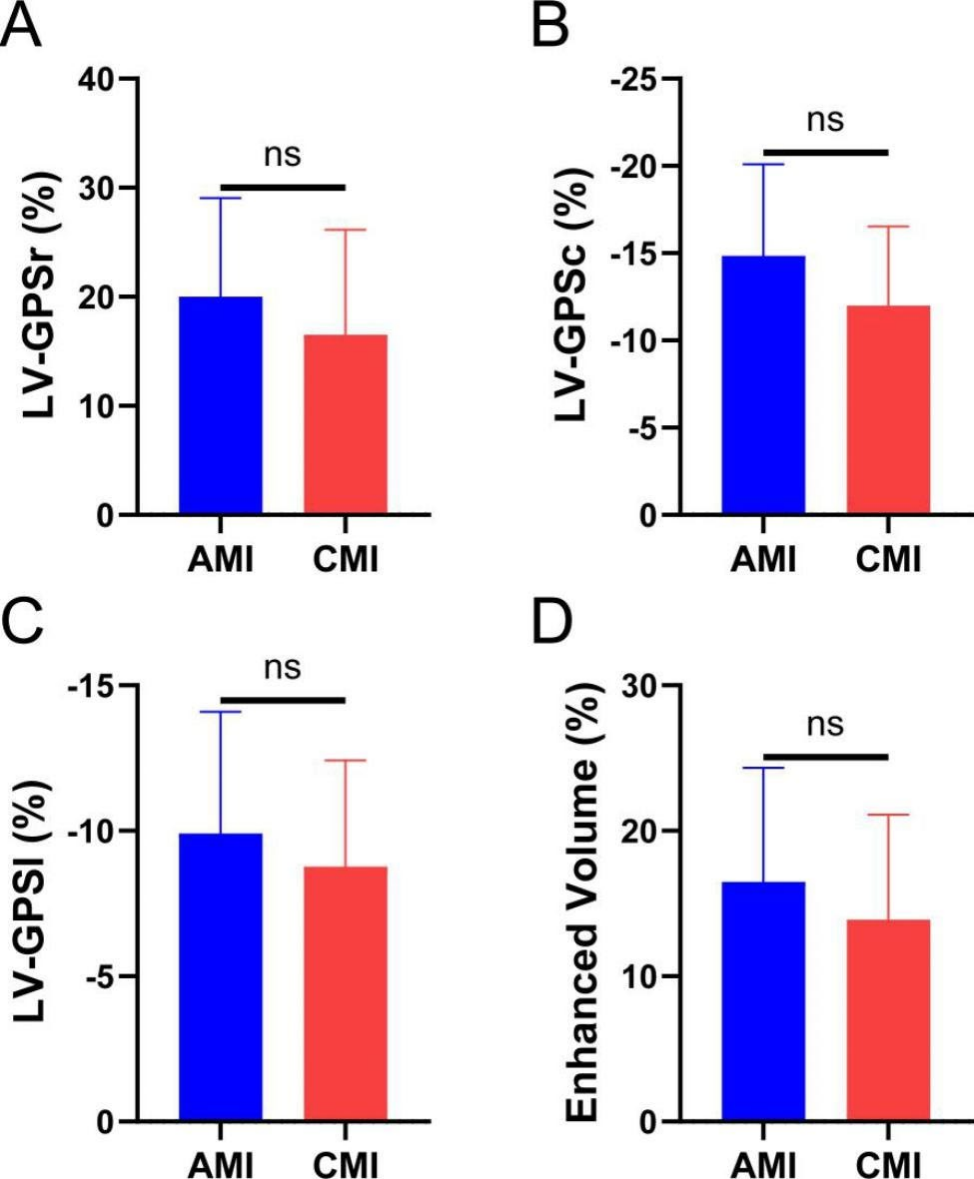
Independent samples t-test was performed for comparisons of LV global peak strain and infarcted segments between the two groups. Ns means No Significance; *P < 0.05; **P < 0.01; *** P < 0.001 ;

As shown in Table 2, patients with CMI had higher LV end-diastolic volume and LV end-systolic volume; and bigger LV mass index than patients with AMI and the controls; controls had greater LVEF than patients with AMI and CMI. No significant difference was noted in LV SV, CO, and CI among these three groups. There was no difference in infarct volume and LV strain between AMI and CMI (Fig. 2).

**Table 2:** LV function and strain parameters

	AMI(n = 36)	CMI(n = 29)	Control(n = 30)	P1 values	P2values	P3values
LV conventional parameters						
LV EDV(ml)	134.95 ± 6.52	194.95 ± 13.42	124.92 ± 7.70	**0.000**	0.623	**0.000**
LV ESV(ml)	71.35 ± 6.79	121.12 ± 13.04	47.09 ± 1.89	**0.001**	0.154	**0.000**
LV SV(ml)	63.59 ± 4.16	73.82 ± 5.00	77.83 ± 7.67	0.339	0.287	0.975
LV EF(%)	48.36 ± 3.32	40.0 ± 3.40	60.81 ± 2.86	0.150	**0.031**	**0.000**
LV CO(l/min)	4.56 ± 0.27	5.23 ± 0.41	5.05 ± 0.45	0.371	0.759	0.852
CI(l/min/m2)	2.58 ± 0.15	2.96 ± 0.22	2.85 ± 0.23	0.329	0.703	0.861
LV Mass(g)	124.88 ± 6.35	159.23 ± 16.05	99.36 ± 5.79	**0.044**	0.135	**0.000**
Infarct volume (%LV)	15.80 ± 8.79	14.26 ± 5.38	-	0.696		
LV strain						
LV-GPSr (%)	20.03 ± 9.025	16.50 ± 9.66	-	0.284		
LV-GPSc (%)	-14.84 ± 5.25	-11.99 ± 4.55	-	0.110		
LV-GPSl (%)	-9.91 ± 4.19	-8.77 ± 3.64	-	0.417		

P1 for AMI versus CMI. P2 for AMI versus normal controls. P3 for CMI values versus normal controls. Bold values indicate statistical significance. There was no difference between AMI and CMI about infarct volume and LV global peak strain

### Left atrial dysfunction

**Fig. 3:**
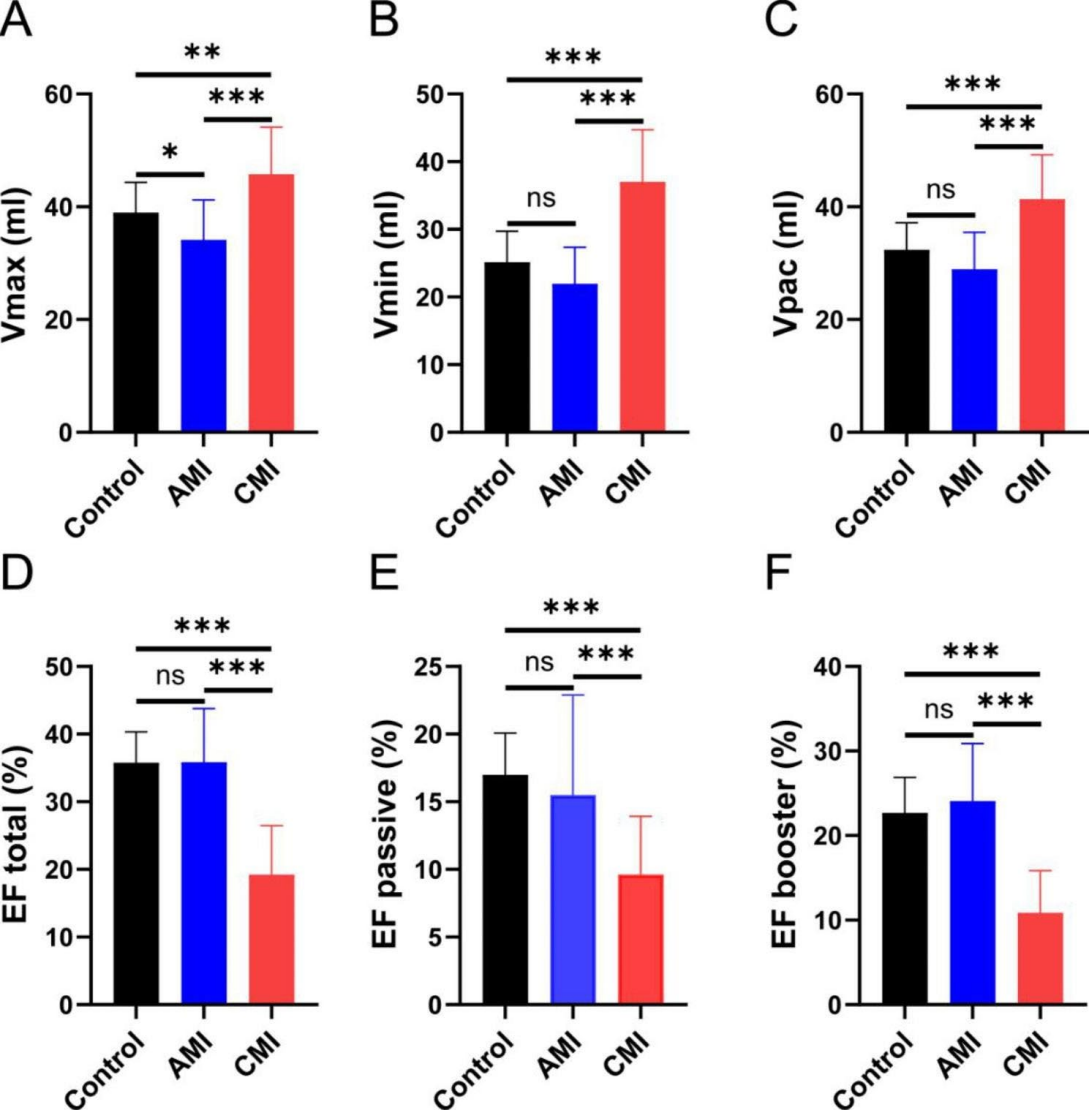
One-way ANOVA with post hoc pairwise comparison test was performed for comparisons of LA function among three groups. Ns means No Significance; *P < 0.05; **P < 0.01; *** P < 0.001

**Fig. 4:**
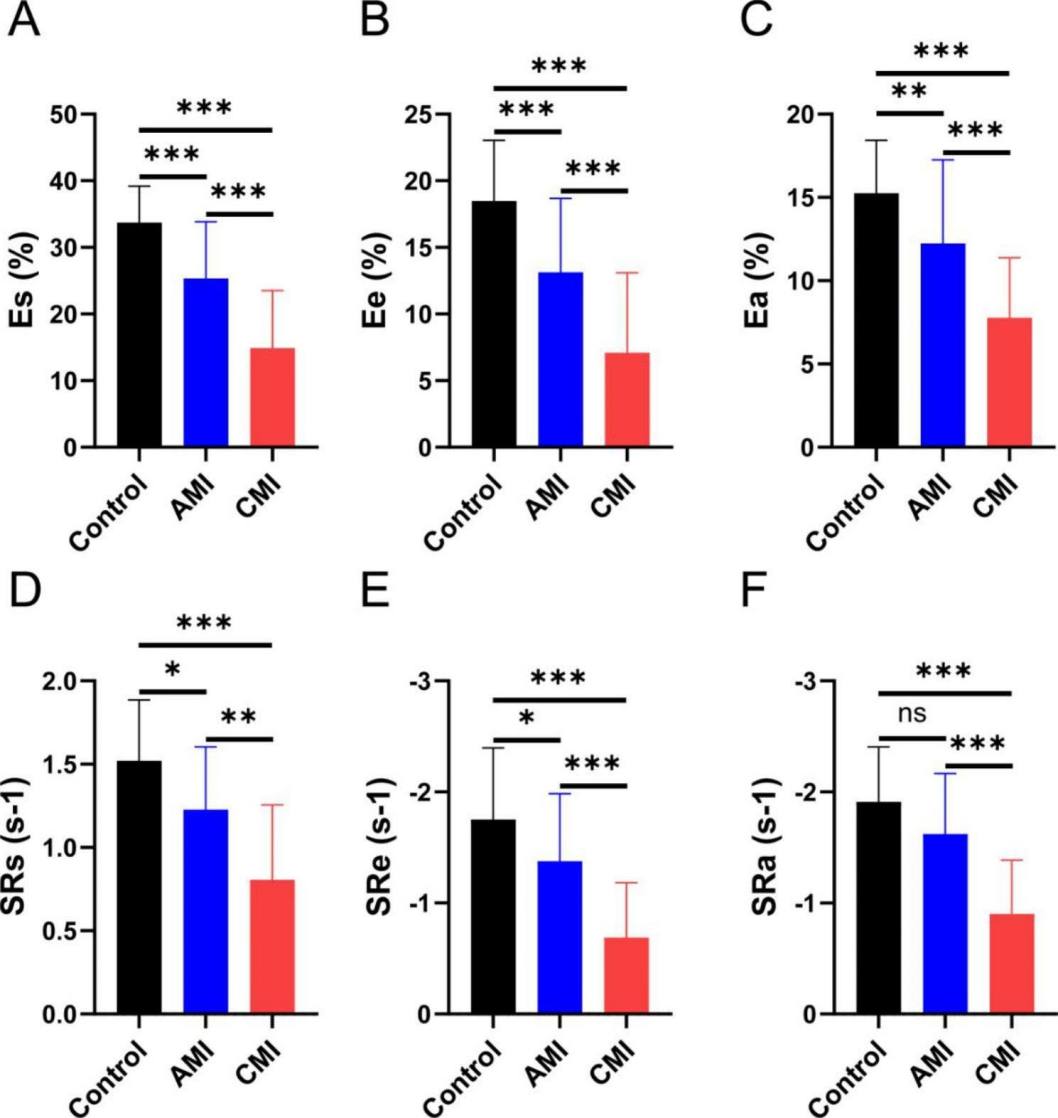
One-way ANOVA with post hoc pairwise comparison test was performed for comparisons of LA strain and strain rate among three groups. Ns means No Significance; *P < 0.05; **P < 0.01; *** P < 0.001

As assessed by volumetric changes and deformation indexes, LA volumes and dynamics were compared among the three groups in Table 3. LA pre-contractile volumes, max- and minimum LA volumes were the largest in CMI, followed by patients in AMI and controls(all *p* < 0.05). The CMI, however, had a lower maximal capacity than acute patients(*p* = 0.047). LA total EF, passive and active EF showed significantly reduced in CMI cases compared to those in AMI and controls(all *p* < 0.01). Left atrial reservoir, conduit functional, and booster pump parameters, including strain and strain rate, showed significantly different among these three groups(all *p* < 0.05) (Figs. 3 and 4).

**Table 3:** LA volumetric and deformation parameters assessed by CMR-FT

	AMI(n = 36)	CMI(n = 29)	Control(n = 30)	P1values	P2values	P3values
LA volumetric parameters						
Vmax, ml	33.95 ± 1.95	46.94 ± 3.0	39.45 ± 1.47	**0.000**	**0.047**	**0.017**
Vpre-a, ml	28.94 ± 1.69	42.36 ± 2.78	32.72 ± 1.32	**0.000**	0.376	**0.003**
Vmin, ml	22.17 ± 1.44	37.92 ± 2.69	25.28 ± 1.26	**0.000**	0.513	**0.000**
LA reservoir function						
EF-total	34.70 ± 1.97	19.36 ± 1.99	36.36 ± 1.19	**0.000**	1.000	**0.000**
εs, %	24.18 ± 1.80	14.27 ± 2.03	34.63 ± 1.33	**0.001**	**0.000**	**0.000**
SRs, s-1	1.23 ± 0.089	0.75 ± 0.10	1.57 ± 0.10	**0.005**	**0.029**	**0.000**
LA conduit function						
EF-passive	14.81 ± 1.41	9.70 ± 1.13	17.16 ± 0.67	**0.011**	0.359	**0.000**
εe, %	13.01 ± 1.38	6.47 ± 1.42	18.89 ± 1.07	**0.004**	**0.003**	**< 0.0001**
SRe, s-1	-1.37 ± 0.156	-0.64 ± 0.10	-1.80 ± 0.15	**0.005**	0.088	**0.000**
LA booster pump function						
EF-active	23.49 ± 1.60	10.78 ± 1.57	23.22 ± 1.15	**0.000**	1.000	**0.000**
εa, %	11.17 ± 0.75	7.79 ± 0.98	15.73 ± 0.87	**0.032**	**0.000**	**0.000**
SRa, s-1	-1.52 ± 0.098	-0.85 ± 0.12	-1.98 ± 0.14	**0.002**	**0.017**	**0.000**

P1 for AMI versus CMI. P2 for AMI versus normal controls. P3 for CMI values versus normal controls. Bold values indicate statistical significance. There was significant difference among three groups about LA strain and strain rate

### Cardiac MR strain parameters for differentiating between AMI and CMI

**Fig. 5:**
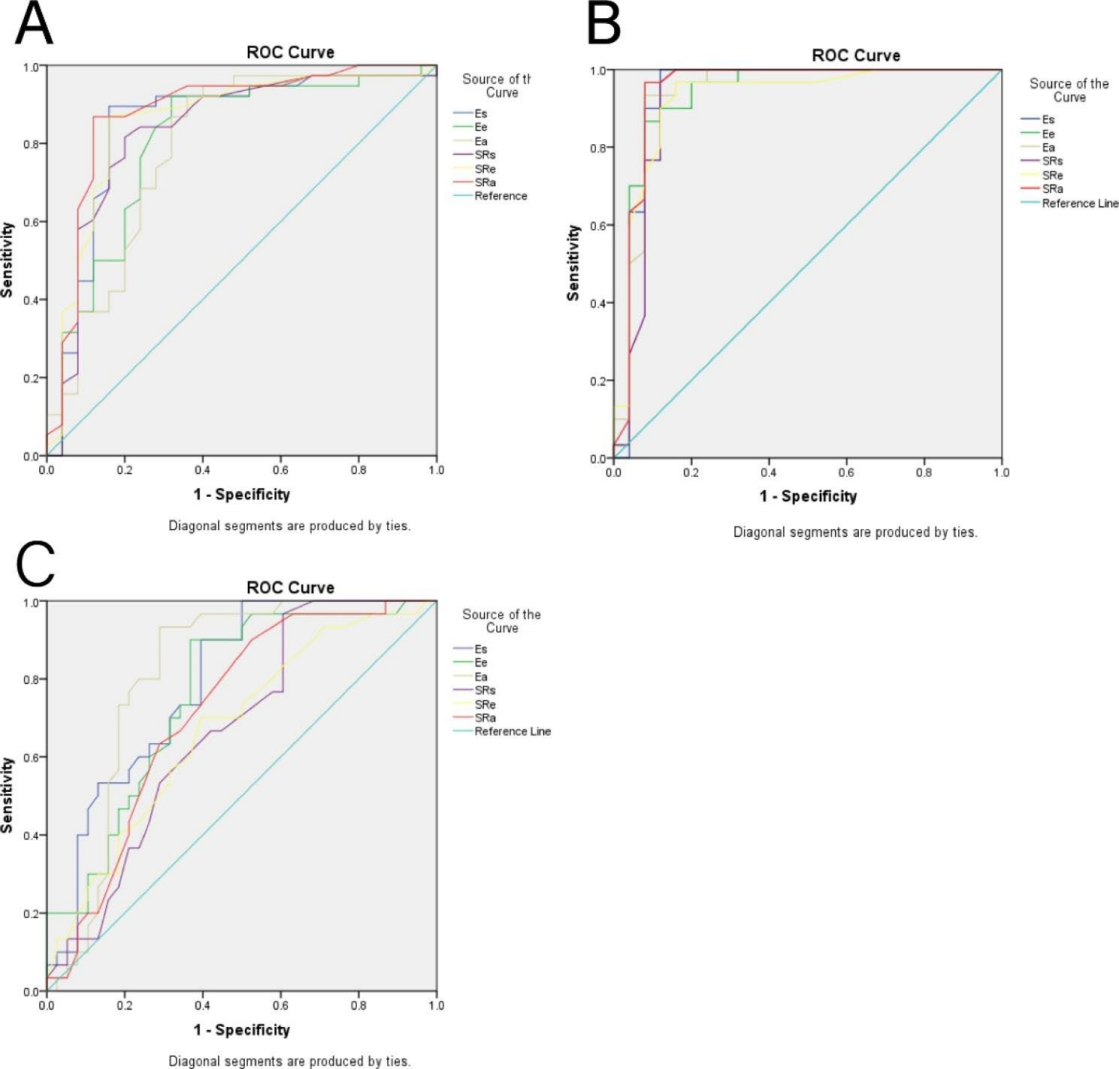
Graph showing results of ROC analysis for differentiation among three groups. (A) The results of ROC analysis for differentiating the AMI and CMI (the AUCs of Es, Ee, Ea, SRs, SRe, SRa were 0.847, 0.805, 0.79, 0.834, 0.857 and 0.879, respectively); (B) ROC analysis for differentiating between AMI and controls (the AUCs of Es, Ee, Ea, SRs, SRe, SRa were 0.789, 0.762, 0.811,0.671,0.673 and 0.717, respectively); (C) ROC analysis for differentiating between CMI and controls (the AUCs of Es, Ee, Ea, SRs, SRe, SRa were 0.941, 0.932, 0.937; 0.924; 0.924 and 0.947, respectively)

Of all the LA strain and strain rate parameters obtained, SRa yielded the best AUC (Fig. 3) of 0.879 for determining between AMI and CMI. When SRa≥-0.817 was taken as the boundary point of diagnosing AMI and CMI, the sensitivity and specificity were 87% and 88%, respectively (Fig. 5).

### Reproducibility

LA volumetric and deformation parameters were reproducible on an intra- and inter-observer level. The CV% and ICC were summarized in Table 4.

**Table 4:** Reproducibility of the LA strain and function analysis by CMR-FT

	Intra-observer		Inter-observer	
	ICC	95% CI	ICC	95% CI
εs, %	0.970	0.783–0.997	0.891	0.447–0.988
εe, %	0.962	0.882–0.988	0.837	0.560–0.947
εa, %	0.959	0.867–0.987	0.883	0.646–0.962
SRs, s-1	0.946	0.840–0.982	0.859	0.578–0.954
SRe, s-1	0.947	0.846–0.982	0.850	0.290–0.959
SRa, s-1	0.949	0.857–0.984	0.846	0.310–0.975
Vmax, ml	0.981	0.942–0.994	0.945	0.832–0.983
Vpac, ml	0.963	0.891–0.988	0.943	0.643–0.994
Vmin, ml	0.972	0.913–0.991	0.937	0.539–0.993
EFtotal, %	0.979	0.937–0.994	0.911	0.718–0.973
EFpassive	0.973	0.917–0.991	0.897	0.714–0.942
EFbooster	0.972	0.908–0.991	0.921	0.765–0.975

LA, left atrial; ICC = intraclass correlation coefficient; CI, confidence interval

## Discussion

The current study investigated the diagnostic accuracy of LA function and strain parameters by CMR-TT for differentiating between AMI and CMI. Our findings demonstrate using quantitative tissue approaches in noncontrast methods can be used to detect the stage of MI. Based on the LA deformation and strain measurement, LA strain and strain rate are the predominant parameters for differentiating AMI from CMI. The results of our study provide several vital advances: (I) there were significant differences in the strain and strain rate parameters among AMI, CMI, and controls; these parameters could be utilized to track the stage of myocardial infarction in MI patients; (II) SRa of LA was the best at differentiating between AMI and CMI, with high diagnostic accuracy in all patients; (III) Patients with acute and chronic myocardial infarction can be identified by LA function parameters; however, there is no difference between the AMI and normal groups; (IV) The left global ventricular strain cannot be utilized the key difference between AMI and CMI. As a result, quantitative measurement of LA strain and strain rate could be a valuable tool for non-invasive evaluation, detection, and differentiation of acute and chronic infarction.

LA function is closely related to changes in overall heart function, which has significance for clinical research. Echocardiographic imaging of myocardial deformation can reveal impairments of LA function. Still, CMR is the “gold standard” for evaluating cardiac morphology and function with high accuracy and repeatability that allows comprehensive evaluation of left atrial structure and function from multiple perspectives [11]. In our cohort, although no differences were noted in LA volumetric parameters between AMI and controls, impaired LA strain is already impaired in AMI patients. There were differences between the three groups in terms of LA strain and strain rate. The area under the ROC curve demonstrates that strain and strain rate have higher diagnostic characteristics for identifying AMI and CMI. The response to an ischemic event has been characterized as a dynamic process. Non-viable infarcted tissue leads to an increase in the cardiac workload of the remaining viable myocardium and subsequently to compensatory hypertrophy [19, 20]. While this compensatory process may be viewed as a positive response to keep blood supply to the systemic circulation, it leads to increasing systolic and diastolic volume [21]. So, we think the impaired LA function may be related to the decreased LV systolic and diastolic function and precede left atrial enlargement and abnormal left longitudinal ventricular function [22]. In terms of LA function, the reservoir and conduit functions contribute the most during early diastole, while the booster pump function is the basis for LV filling during late diastole. Previous studies demonstrated that LA strain could reflect myocardial deformation before the clinically apparent LV functional disorders in AMI [17, 23]. In hypertrophic cardiomyopathy, the impaired LA reservoir strain significantly increases mortality risk and HF development or progression [24, 25]. In the Multi-Ethnic Study of Atherosclerosis (MESA), LA dysfunction preceded HF incidence in the asymptomatic general population, and LA reservoir strain was an independent predictor of HF [26]. Nayyar, D [27] et al. described a compensatory increase in atrial booster pump function in the presence of impaired conduit function after STEMI. LA strain reflects atrial compliance and atrial contractility and relaxation, modulated by the descent of the LV base during systole [28, 29]. Therefore, as an early parameter, LA strain may be more helpful in detecting diastolic alterations before LA enlargement. In this context, it is interesting to speculate that the LA strain obtained from CMR-TT is a potential biomarker for distinguishing AMI from CMI, even though further validation studies are needed.

LA modulates LV diastolic filling and cardiac performance during hemodynamic stress or exertion by reservoir, conduit, and booster pump functions [30]. Preserved LA active function represents a compensatory mechanism to maintain stroke volume and LV filling with mild diastolic dysfunction. Its deterioration reflects the reduction of LA compliance and LV “decompensation” [31]. In this study, AMI patients had lower left atrial volume and ejection fraction than CMI, but there were not statistically different from the normal. After myocardial infarction, the myofibroblasts gradually replaced myocytes, which increased LV stiffness and affected blood flow from the LA into the LV [32]. Within certain limits, contraction of the LA follows the Frank-Starling mechanism, which means that the work of LA contraction depends on the volume before its active contraction preload. LA deformation may be compensation enhanced when the LA preload increases within a certain range [33]. Preserved LA ejection function in AMI patients represents a compensatory mechanism to maintain stroke volume and LV filling with early diastolic dysfunction [34]. CMI may lead to chronic LA myocardial hypoperfusion, which may further impair LA contractility or decrease LA compliance [35].

Left ventricular performance and LVEF are most often quantified to assess cardiac function in cardiovascular diseases [36]. This study revealed that patients with CMI had bigger left ventricular systolic and end-diastolic volumes than patients with AMI and normal controls. However, AMI was not significantly different from the control sample. The remodelling of an ischemic event is characterized by progressive LV enlargement and increased end-diastolic wall stress, which results in a reduced ventricular ejection following a right-ward shift of end-diastolic and end-systolic pressure-volume relations. But these changes are frequently observed late during the illness [37–39]. If the left ventricular ejection fraction is preserved, it may not accurately reflect myocardial function [40].

Myocardial strain analysis has been developed as a more accurate evaluation of myocardial deformation, with the potential to overcome the limitations of EF and contribute to assessing global and regional myocardial deformation during the cardiac cycle [41, 42]. Previous studies affirmed the accuracy and validity of CMR-TT in different patient populations [43, 44], including MI patients. Myocardial ischemia can lead to myocardial fibrosis developed, which makes myocardial motion and strain lower [45]. The global strain is an indicator of whole heart function and is not effective in differentiating the AMI and CMI. However, some studies [38, 46] have demonstrated that local strain parameters can distinguish between acute and chronic infarction. It may be that the study was longitudinal. Our study, on the other hand, was a cross-sectional study. Moreover, the local myocardial strain is influenced by several variables (such as local myocardial infarction degree, infarct area, the presence of MVO, etc.) [47]. Its reproducibility is poor, so it was not examined in our study.

This study’s major limitations might be explained by its small sample size and the individual variation between the participants. Larger samples would still be needed to determine the diagnostic threshold and promote the quantitative diagnosis between AMI and CMI afforded by this technology. Secondly, there is no classification of the duration of CMI. It’s probable that the longer it lasts, the more prominent alterations in left cardiac parameters will be. Thirdly, differences in strain measurements caused by various scan machines cannot be excluded.

CMR-TT-derived LA strain is a potential and robust tool in demonstrating impaired LA mechanics and quantifying LA dynamics, which have high sensitivity and specificity in the differential diagnosis of acute versus chronic myocardial infarction, and its use is thus worth popularizing in clinical application.

## Data Availability

The data underlying this article will be shared on reasonable request to the corresponding author.
